# Focal laser stimulation of fly nociceptors activates distinct axonal and dendritic Ca^2+^ signals

**DOI:** 10.1016/j.bpj.2021.06.001

**Published:** 2021-06-25

**Authors:** Rajshekhar Basak, Sabyasachi Sutradhar, Jonathon Howard

**Affiliations:** 1 Department of Molecular Biophysics & Biochemistry, Yale University, New Haven, Connecticut; 2 Quantitative Biology Institute, Yale University, New Haven, Connecticut

## Abstract

*Drosophila* class IV neurons are polymodal nociceptors that detect noxious mechanical, thermal, optical, and chemical stimuli. Escape behaviors in response to attacks by parasitoid wasps are dependent on class IV cells, whose highly branched dendritic arbors form a fine meshwork that is thought to enable detection of the wasp’s needle-like ovipositor barb. To understand how mechanical stimuli trigger cellular responses, we used a focused 405-nm laser to create highly localized lesions to probe the precise position needed to evoke responses. By imaging calcium signals in dendrites, axons, and soma in response to stimuli of varying positions, intensities, and spatial profiles, we discovered that there are two distinct nociceptive pathways. Direct stimulation to dendrites (the contact pathway) produces calcium responses in axons, dendrites, and the cell body, whereas stimulation adjacent to the dendrite (the noncontact pathway) produces calcium responses in the axons only. We interpret the noncontact pathway as damage to adjacent cells releasing diffusible molecules that act on the dendrites. Axonal responses have higher sensitivities and shorter latencies. In contrast, dendritic responses have lower sensitivities and longer latencies. Stimulation of finer, distal dendrites leads to smaller responses than stimulation of coarser, proximal dendrites, as expected if the contact response depends on the geometric overlap of the laser profile and the dendrite diameter. Because the axon signals to the central nervous system to trigger escape behaviors, we propose that the density of the dendritic meshwork is high not only to enable direct contact with the ovipositor but also to enable neuronal activation via diffusing signals from damaged surrounding cells. Dendritic contact evokes responses throughout the dendritic arbor, even to regions distant and distal from the stimulus. These dendrite-wide calcium signals may facilitate hyperalgesia or cellular morphological changes after dendritic damage.

## Significance

Animals encounter a wide range of noxious stimuli in the natural world. Nociceptive neurons are specialized cells that sense harmful stimuli and trigger avoidance responses. Class IV cells, located under the cuticle in *Drosophila* larvae, are polymodal nociceptors that respond to noxious mechanical, thermal, optical, and chemical stimuli. To investigate the spatial requirements of mechanoreception in class IV neurons, we measured calcium signals evoked by a focused laser beam that creates highly localized tissue damage. We discovered that different cellular compartments—axons and dendrites—responded differentially depending on whether the stimulus makes direct contact with the neuron or not. This provides evidence that mechanical nociception in class IV cells occurs via two distinct pathways.

## Introduction

Nociception is the sensation of painful or injurious stimulation. The peripheral nervous system senses noxious stimuli through nociceptive cells, which signal to the central nervous systems to trigger appropriate behavioral responses (1,2). Although much is known about the molecular basis of thermal, chemical, and mechanical nociception (3,4), many questions remain. For example, injurious mechanical stimuli are difficult to replicate reliably and could have multiple direct effects on the nociceptors or may have indirect effects via damage to cells in the surrounding tissue. Therefore, elucidating the transduction pathways for nociceptive mechanical stimuli is likely to be difficult.

*Drosophila* class IV dendritic arborization (da) neurons are polymodal nociceptors that serve as a model system for studying nociception (5). These highly branched cells (6) innervate the epidermis of the larval body and respond to noxious mechanical (7, 8, 9), thermal (10, 11, 12), chemical (13), and ultraviolet and short-wavelength light (14,15) stimuli. Noxious stimulation triggers avoidance behaviors in the larvae that are attenuated when these cells are specifically ablated (12). A striking ecological example of nociception by this cell is the larval avoidance response to attacks by parasitoid wasps, which puncture the cuticle with their ovipositors to lay eggs in the larvae (16,17). Silencing class IV neurons alone resulted in the loss of defensive rolling escape behaviors (16). The dense network of class IV dendrites, which have a mesh size of several microns (18), may increase the likelihood that an ovipositor, which has a diameter that tapers from 20 *μ*m down to 1 *μ*m (17), makes direct contact with the arbor.

The question we address is how the ovipositor stimulates the class IV cell. It is reported that penetration of the larval cuticle by the wasp’s ovipositor can physically damage the fine dendrites of class IV cells (2). If the damage directly punctures the dendrite’s plasma membrane, this could lead to a local depolarization of the membrane potential, which could propagate electrotonically or by action potentials to the cell body, axon and then to the central nervous system to trigger an escape behavior. Alternatively, it is possible that the ovipositor damages other cells such as epidermal or muscle cells, which in turn signal to the class IV cells via factors released into the extracellular medium or through acidification; these factors or protons could then bind to receptors on the membrane of class IV dendrites, leading ultimately to the opening of ion channels and receptors potentials that propagate to the cell body and axon (2). This indirect pathway would be analogous to the P2X3 receptors of vertebrate nociceptors that bind to ATP released by damaged cells (19,20).

In this study, we investigated potential direct and indirect nociceptive mechanisms using a focused laser beam to locally damage class IV neurons and/or the adjacent tissue. We then used the genetically encoded calcium reporter GCaMP6f (21) to test whether the stimuli evoked calcium responses in class IV cells. We found that stimuli trigger two distinct calcium signaling responses based on their location with respect to the class IV dendrites. If the laser damages the adjacent tissue but not the dendrite, then robust calcium responses are recorded in the axons but not in the dendrites or cell bodies. If the laser damages the dendrites, then robust calcium responses are recorded throughout the dendrite and cell body, in addition to the axon. Thus, class IV cells are excited by both direct and indirect nociceptive pathways.

## Materials and methods

### *Drosophila* strains and husbandry

Fly lines were obtained from the Bloomington *Drosophila* Stock Center (Bloomington, IN) and through generous gifts from Damon Clark (Yale University) and Fernando Vonhoff (University of Maryland, Baltimore County). Fly stocks were maintained at 25°C in a humidity-controlled incubator (60% humidity) on standard apple agar-based food (Archon Scientific, Durham, NC) with 12-h light/dark cycles. Fly crosses were maintained in fly chambers on apple juice agar-based food (mixture of apple agar concentrate, propionic acid, phosphoric acid, and water) with a generous dollop of yeast paste at 25°C and 60% humidity. Larvae 68–72 h after egg laying were used for all imaging experiments. The following fly lines were used to image class IV da neurons: +;*20XUAS-GCaMP6f* ;+ (Bloomington #42747), +;*ppk-Gal4*;+ (Bloomington #32078), +; ;*ppk-CD4-tdTomato* (Bloomington #35845), +;*ppk-CD4-tdTomato*;+ (Bloomington #35844),+; ;*ppk-CD4-tdGFP* (Bloomington #35843).

### Microscopy and imaging experiments

#### Live-cell confocal imaging

Larvae were timed and selected 68–72 h after egg lay (AEL) for imaging. Before imaging, larvae were washed in distilled water and gently rolled on a glass slide with a paint brush to remove excess food and debris. Larvae were then placed on a Cellvis (Mountain View, CA) 35-mm glass-bottom dish (D35-20-1.5-N) and allowed to acclimatize for 60 s. Larvae were then immobilized using a single-layer polydimethylsiloxane (PDMS) device using a protocol as previously described (22). Briefly, larvae were positioned on the center of the dish and gently constrained inside the PDMS cavity. The PDMS was then adhered to the dish by applying slight suction using a 30 mL syringe. No anesthetic was used. Samples were then mounted on the microscope stage, illuminated with Nikon (Tokyo, Japan) lasers (488 or 561 nm at 30–50% laser power), and imaged at 8–10 Hz on a spinning disk microscope: Yokogawa (Tokyo, Japan) CSU-W1 disk (pinhole size 50 *μ*m) built on a fully automated Nikon TI inverted microscope with perfect focus system, a scientific complementary metal–oxide–semiconductor camera (Zyla 4.2 plus sCMOS), and Nikon Elements software with either a 40× (1.25 NA, 0.1615 micron pixel size) or 20× (0.50 NA, 0.3225 micron pixel size). The temperature of the sample region was maintained using an objective space heater at 25°C. Samples were manually focused for each cell before image acquisition. No more than three cells were imaged from an individual larval sample. All data sets represent cells from at least four independent larval samples.

#### Laser stimulation

Stimulation of class IV da neurons was performed using a 405-nm laser (OBIS 405 nm LX 100 mW; Coherent, Santa Clara, CA), which was connected to the microscope through an empty port. Integrated wattage values of the laser were measured using a microscope slide power sensor (Thorlabs, Newton, NJ) at the sample plane. Activation of the laser was synchronized to the imaging rate using a custom LabView macro. Stimulus intensity was user defined before each experiment (10–100%, 0–43 mW integrated power) and administered for 100–200 ms. The precise location of the laser was calibrated using a custom graticule set in NIS Elements (Nikon) and tested before each experiment. For images targeting the soma, the laser was focused on the center of the cell body. Proximal dendrites were stimulated along a main branch 10–30 *μ*m from the cell body. For distal branches, stimulus was administered to a branch 150–200 *μ*m from the soma. Stimulation experiments were performed over 30–45 s, in which the stimulus was administered after 10–12 s of initial baseline recording for each cell.

### Data analysis

#### Image processing

Videos were analyzed using ImageJ (National Institutes of Health, Bethesda, MD). When necessary, videos were stabilized using the Template-Matching or Image Stabilizer plug-ins. For each cell, several regions of interest (ROI) were manually selected for each cell from seven different locations along the entire dendritic tree to study any differential responses within the same cell: soma (one ROI), axon (two ROIs), and dendritic arbors (four ROIs). Care was taken to minimize the background by contouring the ROI to encompass only the cellular region being considered. Corresponding fluorescence values for each ROI were extracted in ImageJ and imported into MATLAB (The MathWorks, Natick, MA). Baseline fluorescence *F*_0_ was calculated as the camera’s mean fluorescence signal for all frames before laser stimulation. The difference in fluorescence from the baseline, (Δ*F*/*F*), was calculated as (*F* − *F*_0_)/(*F*_0_ − 100), where *F* is the fluorescence signal and 100 is the manufacturer’s camera offset. The time series data were median filtered (width 7) to remove outliers resulting from noise or movement. For the measurement of puncture wounds, cells were stimulated, and then *z*-stacks were acquired at 0.5 *μ*m *z*-intervals. Diameters were then analyzed by taking line scans through the center of the wounds on maximal-projection images.

#### Calcium imaging response criteria

ROIs were scored as being responsive to the stimulus if the Δ*F*/*F* at any frame after stimulation was greater than five SDs above the baseline before stimulation. The largest Δ*F*/*F* value for all frames poststimulation was determined to be peak values Δ*F*/*F*. The time point when Δ*F*/*F* was equal to or greater than five SDs above baseline *F* was defined as the latency.

#### Modeling

We modeled the observed dendritic calcium signal magnitudes as a function of intensity (integrated power) and irradiance. We asked the following question: can the overlapping geometry of the stimulus and the dendrite account for the observed differences in responses to narrow and wide stimulus profiles and when applied to thick, proximal dendrites and thin, distal dendrites? The laser was modeled as a two-dimensional Gaussian with experimentally measured variance; the spatial profile of the laser was measured using interference-reflection microscopy (23) by analyzing the reflection of the laser on a coverslip and using a line scan in ImageJ. A Gaussian was fit over the line scan in MATLAB to compute the SD, *σ*. To test whether the observed laser-activated calcium responses are due to surface or volume illumination, we considered two different models. First, we considered overlap of the laser profile with the cylindrical surface of the dendrite (Eq. 1). In the second model, we considered overlap of the laser profile with the cylindrical volume of the dendrite (Eq. 2).

(1) $$F_{\text{s}}=\gamma \overset{R}{\underset{-R}{\int }}\overset{+\infty }{\underset{-\infty }{\int }}{\left(\frac{P}{2\pi \sigma^{2}}\right)}^{n}{\left(e^{\frac{-x^{2}-y^{2}}{2\sigma^{2}}}\right)}^{n}\cdot 2\sqrt{1+\frac{x^{2}}{R^{2}-x^{2}}}\;dx\;dy,$$

(2) $$F_{\text{v}}=\gamma \overset{R}{\underset{-R}{\int }}\overset{+\infty }{\underset{-\infty }{\int }}{\left(\frac{P}{2\pi \sigma^{2}}\right)}^{n}{\left(e^{\frac{-x^{2}-y^{2}}{2\sigma^{2}}}\right)}^{n}\cdot 2\sqrt{R^{2}-x^{2}}\;dx\;dy.$$

Here, *F*_s_ and *F*_v_ are the theoretical peak values of Δ*F*/*F* corresponding to each model, *P* is the integrated laser power, *σ* is the SD of the Gaussian, and *R* is the radius of the dendrite. The soma was modeled as a cylinder with radius *R*_soma_ = 1 *μ*m significantly larger than that of proximal (0.5 *μ*m) and distal dendrites (0.2 *μ*m) (24). This simplification is justified as the laser profile dies off exponentially, and *σ* ≪ *R*_soma_. The variable *n* is a free parameter introduced to account for the observed nonlinearity in experimental values, and *γ* is a free parameter corresponding to a conversion factor between units.

*P* ranged between 0 and 100 based on the power output of the laser. *σ* was set at 212 or 425 nm, corresponding to the two different stimulation irradiance settings. Because peak values of Δ*F*/*F* exhibited unequal variances (heteroskedasticity) across the range of stimulation wattages, we computed a set of weights for use in our weighted least-squares fitting by performing a linear regression between the Δ*F*/*F* and the experimental standard deviation of the mean. A detailed table of input values for the models can be found in the Supporting materials and methods. MATLAB’s *fminsearch* was used to compute the values for *n* and *γ* that simultaneously minimized the sum of the squared errors between all theoretical and experimental values. Minimization was performed by considering data from axon ROIs and nonaxon ROIs separately. The surface and volume model were each fit to the data.

#### Statistical analysis

Sample sizes are listed for each data set on the corresponding plots. Capitalized “N” indicates the number of larvae; lowercase “n” is the number of neurons. Statistical analysis was performed in Prism 8 (GraphPad Prism). Sidak’s test was used when making multiple pairwise comparisons. One-way ANOVA was used to determine if statistically significant differences existed between the means of three or more independent groups. For plots showing peak values of Δ*F*/*F*, all data points (open circles) and experimental means (lines) are shown on graphs to demonstrate experimental variability. For plots showing latency, experimental means and SD are shown. Significance was evaluated at *p* < 0.05.

## Results

### Focal 405-nm stimulation triggers cuticular damage, behavioral responses, and intracellular calcium increases

**Figure 1:**
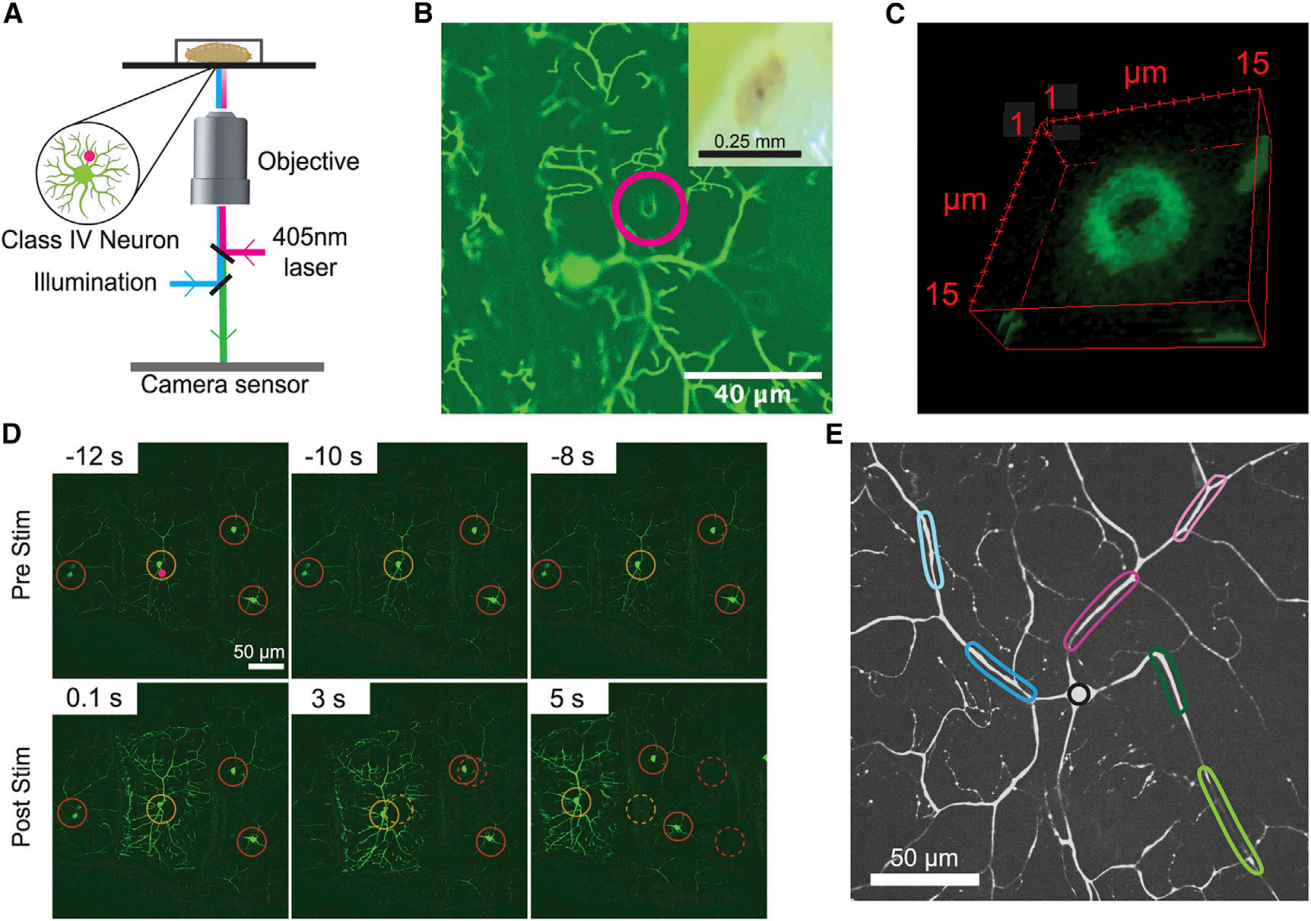
Focused 405-nm laser stimulation triggers larval behavioral responses and calcium signals in class IV cells. (*A*) Schematic diagram depicting the stimulation and imaging setup. (*B*) Example of a puncture wound (*magenta circle*) in the dendritic arbor of a class IV neuron expressing GFP. Inset shows a melanotic spot at the site of illumination. (*C*) The *z*-stack slices of the puncture wound. (*D*) Montage depicting behavioral and calcium responses to 405-nm stimulation at 80% stimulation intensity. Top row shows larva stationary before stimulation; bottom row shows tissue movement and calcium increase measured using the genetically encoded calcium reporter GCaMP6f. Dashed circles indicate the positions of the cell bodies before stimulation. (*E*) Image of a class IV neuron depicting seven regions of interest (ROI): four on dendrites (*magenta* and *blue*), two on the axon (*green*), and one on the soma (*black*). Darker color is more proximal. To see this figure in color, go online.

To study nociception by class IV neurons, we used a focused 405-nm laser beam to mimic penetration of the larval cuticle by the wasp ovipositor. We irradiated individual class IV neurons in unanesthetized larvae that had been constrained in a PDMS device (22) mounted on the stage of a spinning disk confocal microscope (Fig. 1 *A*). When focused to a diameter of 0.5 or 1 *μ*m (full width at half maximum, FWHM), laser illumination with integrated power ≥80% (≥32 mW, Fig. S1) and duration 0.2 s produced cuticular puncture wounds (Fig. 1 *B*), severed dendrites, and caused bleaching that did not recover over 20 min (Fig. S2*, A and B*). The diameters of the puncture wounds were 2–4 *μ*m (Fig. 1 *C*), similar to the diameters of wasp ovipositor barbs, though smaller than the maximal 20-*μ*m diameter of the ovipositor itself (17). These laser powers produced “melanotic spots” (inset to Fig. 1 *B*), a characteristic of cuticular penetration by the ovipositor (17,25). Integrated powers ≤40% did not produce punctures; at these intensities, bleaching of illuminated dendrites occurred but recovered over 20 min (Fig. S2*, C and D*).

Constrained larvae pulsed with the 405-nm laser at ≥80% power for 0.1 s exhibited behavioral responses that manifest as tissue movements (Fig. 1 *D*; Videos S1 and S2). Unconstrained larvae writhed, crawled, and turned upon laser irradiation, with higher stimulation intensity eliciting a stronger response. The laser stimulation was not lethal; all six larvae (70 h AEL) subject to ≥80% maximal power survived for 24 h.

Video S1. Example of a behavioral response (writhing) after laser stimulation

Video S2. Example of a behavioral response (crawling) after laser stimulation

Focal laser stimulation within the dendritic fields of class IV neurons induced intracellular calcium increases. After pulsed stimulation for 0.1 s at 80% power, the fluorescence of the calcium indicator GCaMP6f, expressed specifically in class IV cells (see Materials and methods), increased (Fig. 1 *D*). The fluorescence increases could be observed in the cell body, the dendrites, and the axon, with amplitudes up to severalfold above baseline and lasting for several seconds (Videos S1 and S2). The fluorescence change was mediated, at least in part, by calcium influx through voltage-gated calcium channels: RNA interference of the Ca-*α*1D subunit of voltage-gated calcium channels in class IV neurons resulted in smaller fluorescence changes (Fig. S5), as has been found for thermal responses in these cells (11). Thus, our focused 405-nm laser stimulus is a nonlethal nociceptive stimulus that mimics cuticle penetration by an ovipositor barb, producing both behavioral and cellular responses. The laser stimulus has advantages over an attack by a wasp’s ovipositor, as its position, intensity, geometry, and duration can be controlled precisely.

### The calcium response depends on the position of the 405-nm illumination

**Figure 2:**
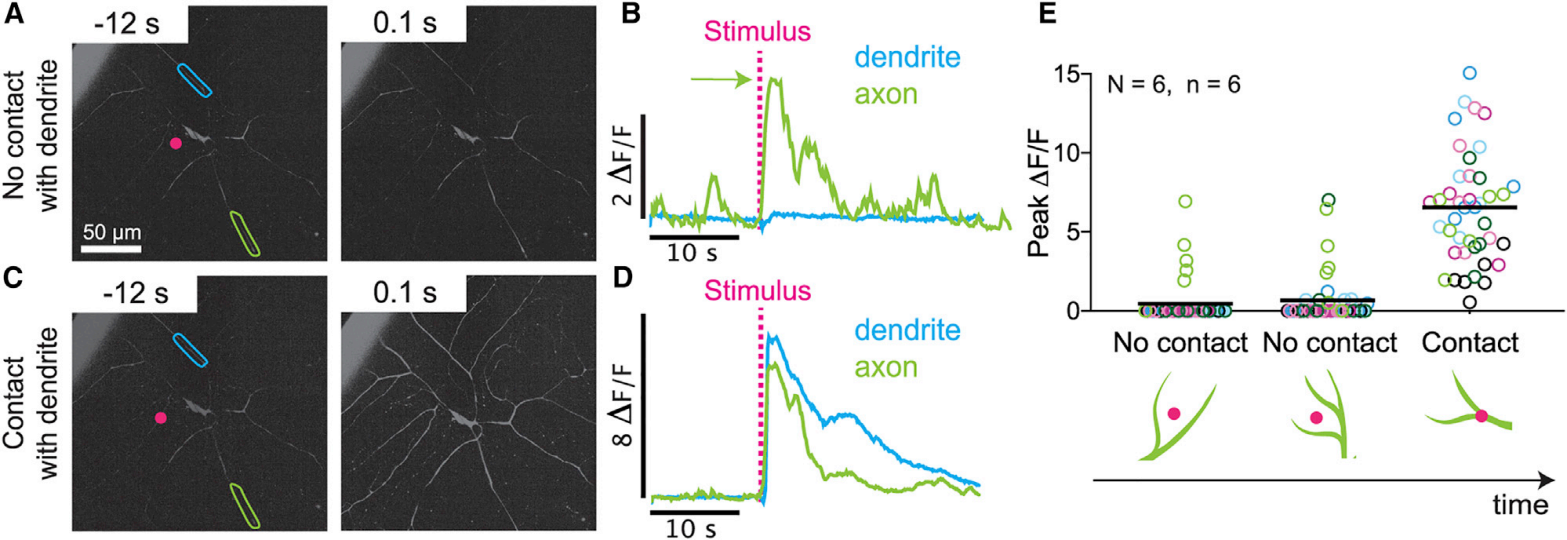
405-nm stimulation triggers two distinct calcium signaling responses in class IV neurons. (*A* and *B*) When the laser is focused adjacent to but not on a dendrite (noncontact), large calcium changes are recorded in the axon (*green* ROI), and only very small changes are recorded in the dendrite (*blue* ROI). (*C* and *D*) When the laser is focused on a dendrite (contact), large calcium changes are recorded in both the axon (*green* ROI) and dendrite (*blue* ROI). (*E*) Magnitude of normalized fluorescence responses across all seven ROIs (*open circles* color coded as in Fig. 1*E*) for a cell stimulated three consecutive times at different locations. The first two stimulations did not make contact with the dendritic arbor and evoked axonal responses only (*green*); the third stimulation made contact and evoked responses everywhere. Black lines indicate means for all ROIs combined. N represents the number of larvae; n represents the number of cells. To see this figure in color, go online.

To test whether physical damage to class IV dendrites is necessary for nociceptive responses, we took advantage of the narrow spatial profile of our laser probe as well as our ability to precisely control its position relative to the dendritic processes. We found that larval behavioral responses were triggered irrespective of the stimulus location. However, we observed different calcium responses in class IV neurons depending on whether the stimulus made direct contact with the dendritic arbor or not. The background fluorescence of the GCaMP6f-expressing cells was sufficiently high to unambiguously identify even distal dendritic processes (e.g., Fig. 1 *E*). We found that noncontact illumination generated calcium transients in the axons of class IV neurons but greatly attenuated signals in the dendrites (Fig. 2*, A and B*; Video S3). In contrast, direct contact of the stimulus with the arbor results in calcium responses throughout the entire cell (Fig. 2*, C and D*; Video S4). To confirm this result, we repeated the same experiment by stimulating single cells three times in three different places; the first two stimuli made no contact with class IV arbors, whereas the third made contact. We found that only axons responded when no contact was made, whereas axon, dendrite, and soma all responded when contact was made (Fig. 2 *E*). Thus, there are two distinct calcium signaling responses: 1) a “noncontact” response in axons only and 2) a “contact” response in all compartments.

Video S3. Non-contact axon response with 80% laser stimulation

Video S4. Contact dendrite response with 80% laser stimulation

### The noncontact response

**Figure 3:**
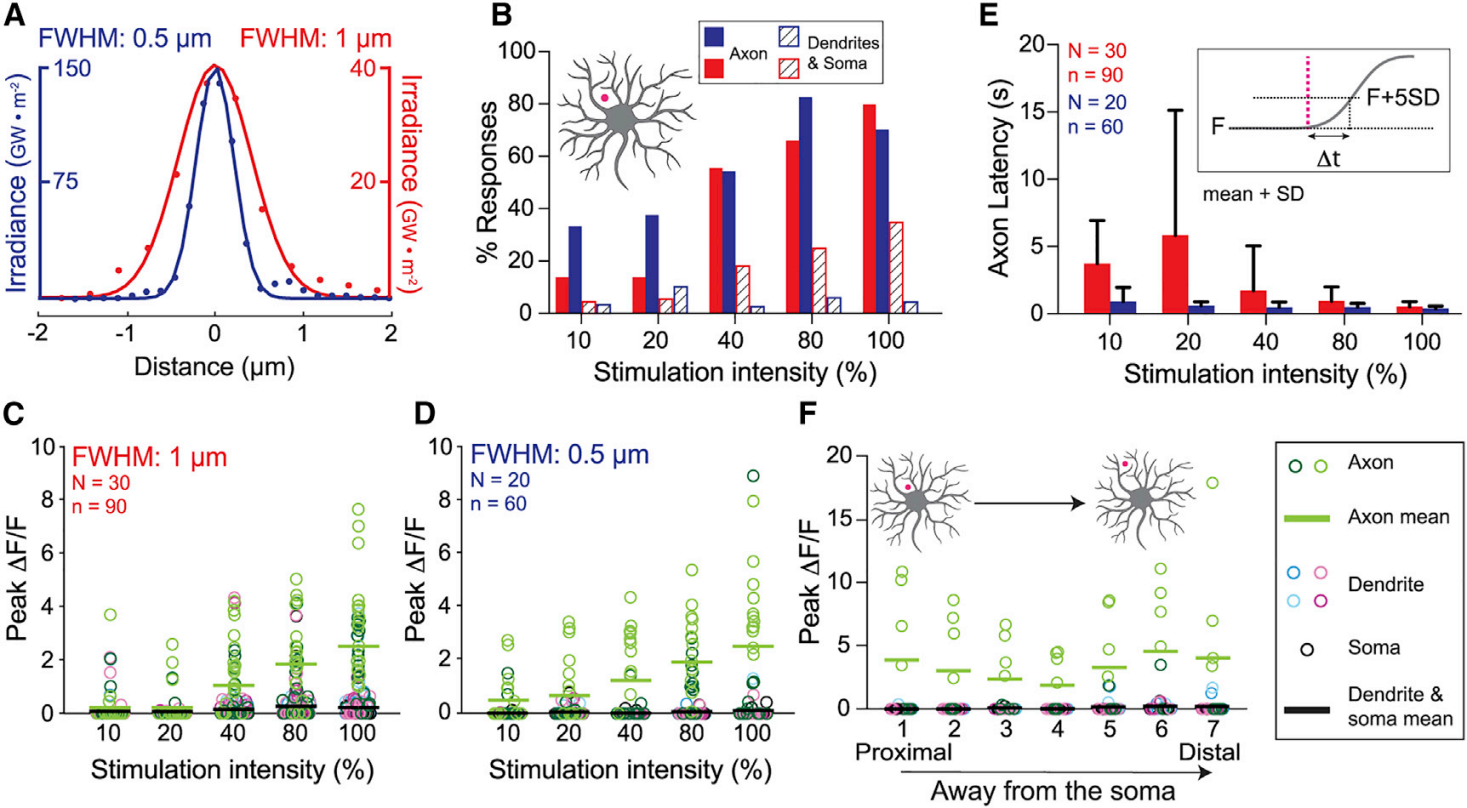
Characterization of the “noncontact” axonal calcium response. (*A*) Line scans of the spatial profiles of the narrower 405-nm profile (0.5 *μ*m FWHM, *blue*) and the wider profile (1 *μ*m FWHM, *red*). When they have the same total power (intensity), the irradiance (on the *y* axes) of the narrower profile is four times larger. (*B*) Frequency of calcium transients in the two axonal ROIs (*solid bars*) and the five dendritic and somal ROIs (*striped bars*) in response to noncontact stimulation across a range of intensities (10–100%). Red and blue correspond to wider and narrower profiles. (*C* and *D*) Peak Δ*F*/*F* values for cells stimulated with no contact. Open circles indicate ROIs color coded as in Fig. 1*E*. Lines denote means of axon ROIs (*green*) and dendrite/soma ROIs (*black*). Statistical comparisons for these data are in Table S6. (*E*) Axonal response latencies. Red and blue histograms correspond to wider and narrower stimuli. Inset: the latency is defined as the time when Δ*F*/*F* = *F* + 5 SD. Statistical comparisons for data are shown in Table S7. (*F*) Peak values of Δ*F*/*F* for seven consecutive noncontact stimuli (0.5 *μ*m FWHM) at increasing distances from the cell body. Ordinary one-way ANOVA test shows no difference between axon means (*p* = 0.8501). N represents the number of larvae; n represents the number of cells. To see this figure in color, go online.

To investigate the conditions under which the noncontact response in axons is triggered, we probed larvae with laser stimuli of different intensities (up to 100% laser power of 45 mW) and spatial profiles (FWHM equal to 0.5 or 1 *μ*m; Fig. 3 *A*). We first asked how the likelihood of a calcium response depended on the light intensity. An ROI (defined in Fig. 1 *E*) was deemed responsive if the relative change in fluorescence, Δ*F*/*F*, after stimulation was larger than five SDs of the baseline fluorescence, *F*, before stimulation (Materials and methods). We found that the percentage of axonal ROIs that responded to noncontact stimuli increased from 10% (FWHM 1 *μ*m) and 30% (FWHM 0.5 *μ*m) at 10% laser power to 70–80% at 100% laser power (Fig. 3 *B*). Thus, the noncontact stimulus reproducibly evokes responses from class IV axons, with narrower profiles giving larger responses at lower total intensities.

We found that the magnitudes of calcium responses were also graded with stimulation intensity. The peak value of Δ*F*/*F* in the axon after the laser pulse increased from an average of 0.2 (FWHM 1 *μ*m) and 0.5 (FWHM 0.5 *μ*m) at 10% laser paper to an average of ∼2.5 at 100% laser power for both stimulation profiles (Fig. 3*, C and D*). Thus, axonal calcium transients induced by noncontact stimulation are not all-or-nothing but rather graded with stimulus intensity.

In contrast to the axonal responses, only a small fraction of dendritic and somal ROIs responded to noncontact stimulation (Fig. 3 *B*, striped bars). Furthermore, the magnitudes of these responses were small: the average Δ*F*/*F*, was ≪1, with few ROIs giving Δ*F*/*F* > 0.1, even at the highest intensities (Fig. 3*, C and D*, *black lines*).

The latencies of the noncontact axonal responses, defined in the inset to Fig. 3 *E*, decreased with increasing intensity (Fig. 3 *E*). The narrower stimulus (0.5 *μ*m FWHM) gave shorter latencies than the wider stimulus. For example, the latencies at 100% power were 0.39 ± 0.17 s (mean ± SD, n = 12 cells) for 0.5 *μ*m FWHM and 0.52 ± 0.38 s (mean ± SD, n = 18 cells) for 1 *μ*m FWHM.

The noncontact axonal response did not depend on the proximity of the stimulus to the cell body or the axon. To test this, we stimulated cells seven times at 80% intensity, with each stimulus progressively further away from the soma. We found no consistent effect of stimulus location (Fig. 3 *F*) (ordinary one-way ANOVA; *p* = 0.8501 was not significant).

In summary, the noncontact axonal response is graded, with higher intensities leading to a higher likelihood of responding, a larger fluorescence change when responding, and a shorter latency. By contrast, dendrites and soma responded infrequently to noncontact stimulation, and the responses were much smaller.

### The contact response

**Figure 4:**
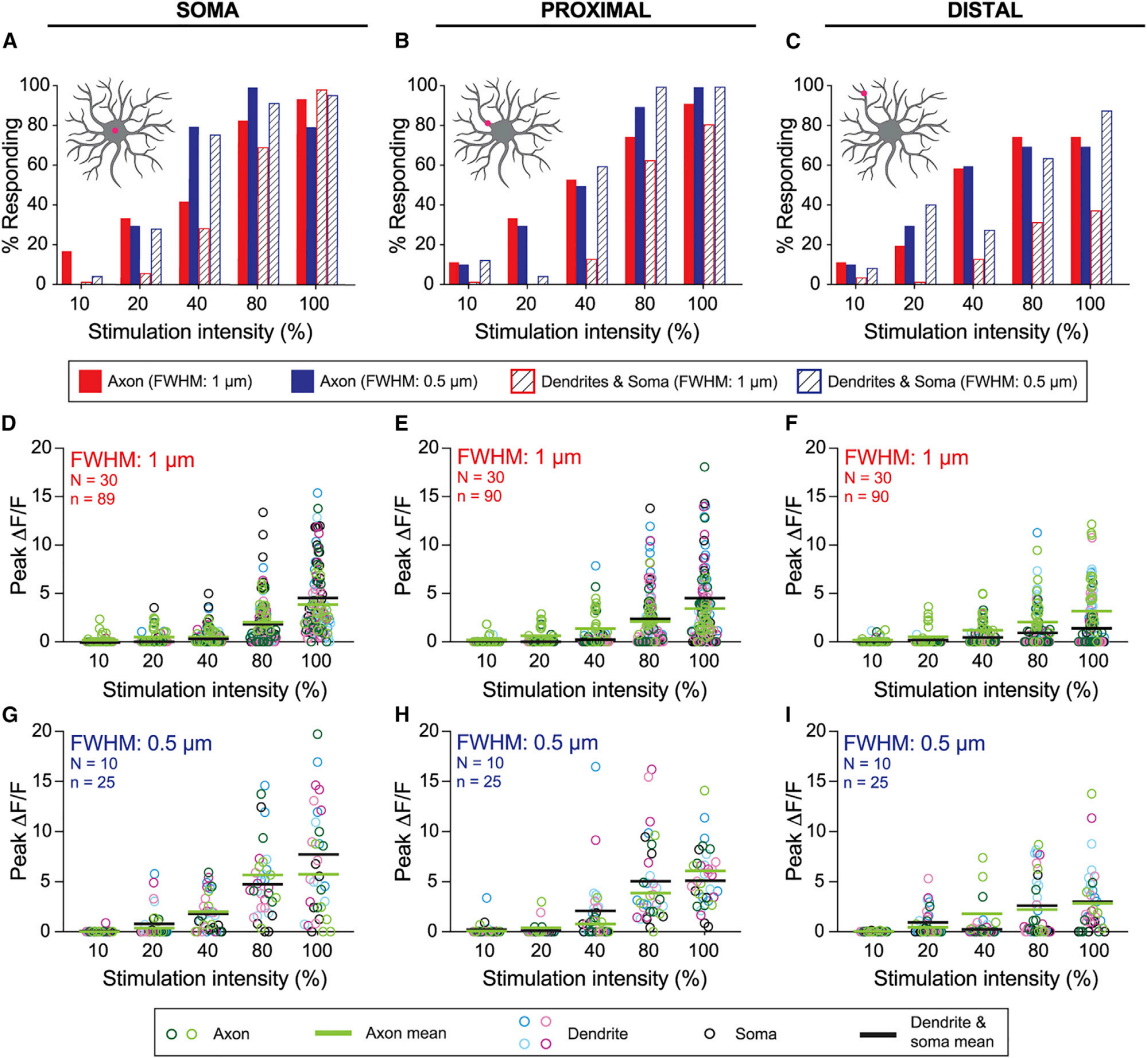
Characterization of the “contact” axonal and dendritic calcium response. (*A*–*C*) Fraction of cells responding to direct stimulation to the cell body (*A*), proximal dendrite (*B*), and distal dendrite (*C*). (*D*–*I*) Peak calcium responses in cells stimulated on the cell body (*D* and *G*), on proximal dendrites (*E* and *H*), and on distal dendrites (*F* and *I*). N represents the number of larvae; n represents the number of cells. Statistical analysis of these data are contained in Tables S4 and S8. To see this figure in color, go online.

To investigate the conditions under which the contact response is triggered, we illuminated the class IV cells directly with the focused laser in three different locations: soma (Fig. 4 *A*), proximal dendrites (Fig. 4 *B*), and distal dendrites (Fig. 4 *C*). To quantify the likelihood of responses for each stimulation condition, we computed the percentage of the two axonal ROIs and the five dendrite and soma ROIs that responded to stimuli of different intensities and spatial profiles. We found that direct stimulation of the soma, proximal dendrites, and distal dendrites all evoked calcium transients throughout the cell with the percentage responding increasing with increasing stimulus intensity (Fig. 4*, A–C*). At the highest intensities, most ROIs responded, with the soma and proximal stimulation being somewhat more efficacious than distal illumination. In contrast to noncontact stimulation where axons respond more frequently over the whole range of stimulation intensities (Fig. 3 *B*), contact stimulation leads to a similar percentage of axon and dendrite calcium responses at higher stimulation intensities (Fig. 4*, A–C*). Interestingly, the magnitude of axonal responses are generally the same for noncontact and contact pathways (Tables S1–S3); only at the highest stimulation intensities (100%) and for the narrower stimulus (FWHM: 0.5 *μ*m) directed at the soma (Fig. 4 *G*; Table S1) and proximal (Fig. 4 *H*; Table S2) dendrites is the response larger in the “contact” response.

The magnitudes of the calcium responses (peak Δ*F*/*F*) increased with increasing stimulus intensity at all locations (Fig. 4*, D–I*; Figs. S3 and S4; Videos S5 and S6). The average response magnitudes for somal and proximal stimulation were similar (Fig. 4*, D, E, G, and H*) and somewhat larger than for distal stimulation (Fig. 4*, F and I*). The average axonal response magnitudes were similar to the average somal and dendritic magnitudes (*green and black lines* in Fig. 4*, D–I*).

Video S5. GCamp6f response at 40% laser stimulation

Video S6. GCamp6f response at 80% laser stimulation

The frequency (Fig. 4*, A–C*) and magnitudes (Fig. 4*, D–I*, *green circles and bars*) of the peak axonal responses were independent of the stimulus profile (Fig. 4*, D–I*, *green circles and bars*). However, the frequency (Fig. 4*, A–C*) and magnitudes (Fig. 4*, D–I*, *colored circles and bars*) of the dendritic and soma responses were significantly larger for the narrower profile. See Table S4 for statistical analysis. We will return to the question of how the stimulus and dendrite geometry effects the responses.

**Figure 5:**
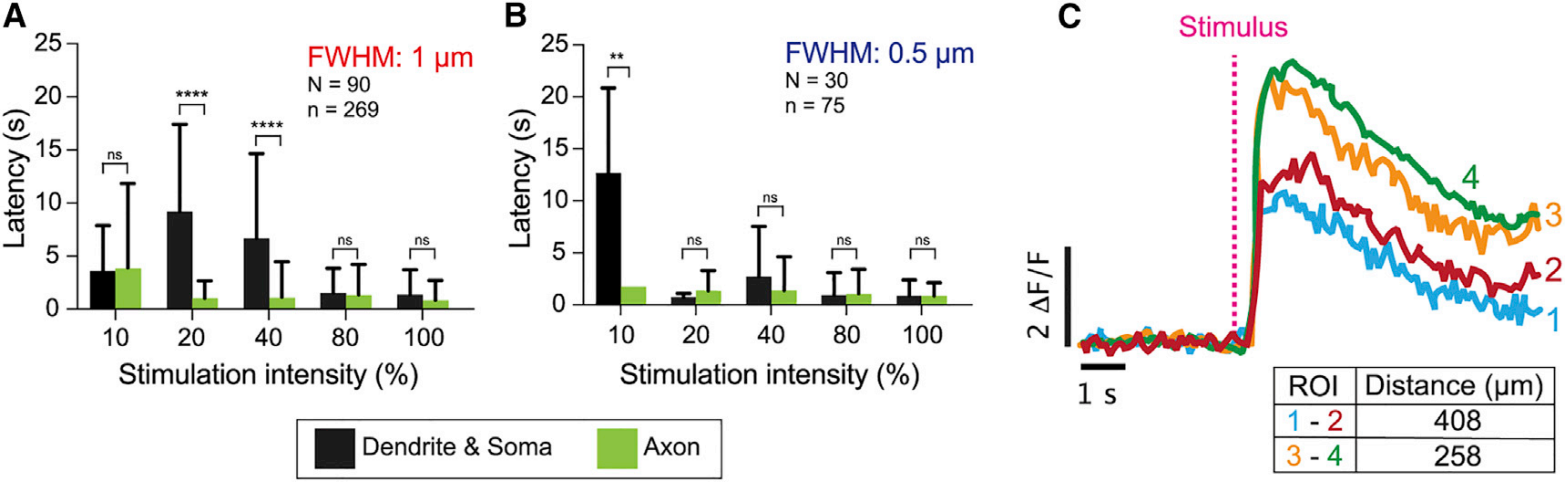
Latencies of axonal and dendritic responses to “contact” stimulation. (*A* and *B*) Latencies for cells stimulated with wider (*A*) and narrower (*B*) profiles at varying intensities (10–100%). Error bars are standard deviations. ns denotes not significant at the 5% confidence level (*p* > 0.05), (∗∗) denotes *p* < 0.01, and (∗∗∗∗) denotes *p* < 0.0001 (Sidak's multiple comparisons test). For statistical comparisons across two different irradiance settings, see Table S9. (*C*) Representative traces from one neuron showing that dendritic regions >250 *μ*m apart on opposite sides of the cell body respond simultaneously, though with a lag relative to the stimulus. See Videos S4, S5, and S6. N represents the number of larvae; n represents the number of cells. To see this figure in color, go online.

The latencies of the contact responses were shorter in the axons than the dendrites (Fig. 5*, A and B*); in other words, the dendritic response rises with a larger delay than the axonal response. For both axons and dendrites, higher intensities gave shorter latencies. The latencies of axonal contact responses were similar to those of noncontact responses (Fig. 3 *E*). Interestingly, the rising phases of the dendritic responses were almost simultaneous in all the dendritic regions, being within the 100-ms frame time of the camera, even though the latency was significantly longer (≥500 ms). For example, directly stimulating a peripheral dendrite gave a response in the same dendrite and in a dendrite on the other side of the cell body (>200 *μ*m distance away) with a time course that rose within 100 ms of each other (one frame) (Fig. 5 *C*). This shows that the dendritic signals propagate at speeds >2 mm/s (= 200 *μ*m/100 ms).

### Contact responses depend on the overlap between the stimulus and the dendrite

**Figure 6:**
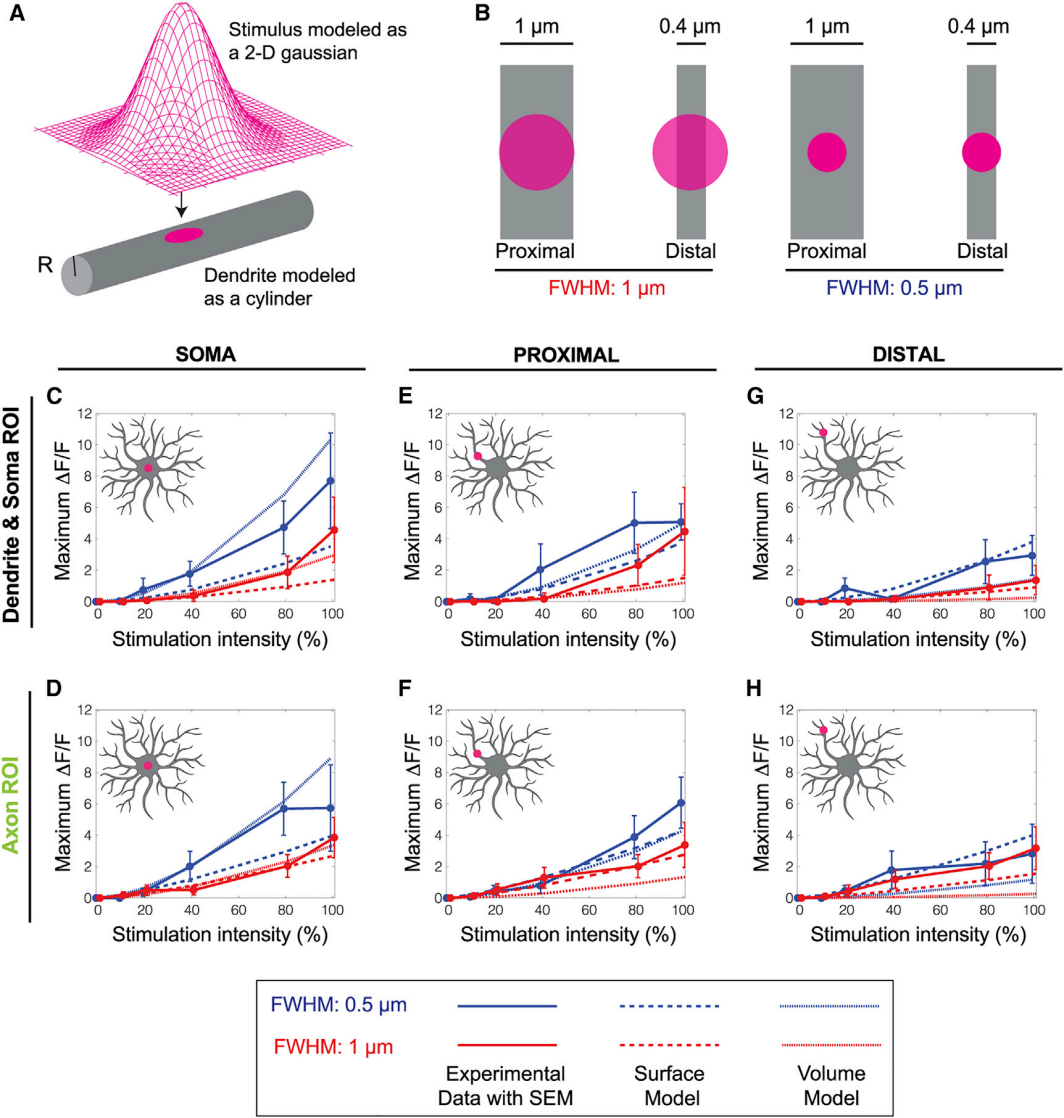
Overlap model for the effectiveness of stimuli in generating responses. (*A*) Schematic of the overlap model: the laser profile is approximated by a two-dimensional Gaussian and the dendrite modeled by a cylinder with radius *R*. (*B*) Top-down view of the two laser profiles projected onto proximal and distal dendrites. Proximal dendrites have radius 500 nm, and distal dendrites have radius 200 nm. (*C*–*H*) Theoretical curves (*lines*) superimposed on the measured peak Δ*F*/*F* for somal (*C* and *D*), proximal dendrite (*E* and *F*), and distal dendrite (*G* and *H*) stimulation. Dashed lines represent the surface model, and dotted line represents the volume model. Model parameters are listed in Tables S10 and S11. To see this figure in color, go online.

The diameters of dendrites decrease from ∼1 *μ*m for the most proximal to ∼0.25 *μ*m for the terminal dendrites (24). Therefore, both the narrower and wider stimuli will fall mostly within the proximal dendrites (and soma), whereas the wide stimulation will fall mostly outside the distal dendrites. Thus, wider profiles are expected to be less effective when applied distally. To test whether this accounts for the differences between proximal and distal stimulation (e.g., Fig. 4*, F and I*), we formulated a mathematical model that takes into consideration both the shape of the laser stimulus and the geometry of the dendrites. The laser beams were modeled as two-dimensional Gaussians with SDs corresponding to the measured FWHMs (Fig. 6 *A*). The dendrites were modeled as cylinders with the estimated diameters 1 *μ*m proximal and 0.4 *μ*m distal (Fig. 6 *B*). The soma was modeled as a cylinder with diameter 2 *μ*m. We accounted for possible nonlinearity between stimulus intensity and response magnitude by introducing an exponentiation factor with exponent (*n*). We then asked whether the observed peak values of Δ*F*/*F* (replotted in Fig. 6*, C–H*) are consistent with the differing overlaps between the stimulus and the dendrite. In other words, does stimulation of thinner dendrites result in smaller responses because a smaller fraction of stimulus is actually making contact with the process? We considered two cases: 1) the response depends on the overlap of the stimulus with the dendrite volume, and 2) the response depends on the overlap of the stimulus with the dendrite surface area. The equations are in the Materials and methods.

We fit the models to the data (Fig. 6*, C–H*) using the measured profiles, dendrite diameters, and stimulus intensities. For each model (volume, surface area) and data set (axon, dendrite/soma), we found the values of the exponent (*n*) and a conversion factor (*γ*), which simultaneously minimized the sum of the least-squares difference between the model and all six associated experimental curves. The models recapitulated the nonlinear increase in response with stimulus intensity, the observed smaller peak values of Δ*F*/*F* for distal stimulation, and the dependence on the profile widths. Interestingly, we found that the axonal responses were a better fit to the surface model than the dendritic and somal responses. Thus, the responses depend on the overlap of the stimulus with the dendrite (Table S5).

## Discussion

In this study, we developed a nonlethal, tunable, in vivo assay for larval nociception using a 405-nm laser that causes highly localized puncture wounds to the larval cuticle. This stimulation evoked behavioral responses similar to nociceptive avoidance responses triggered by a wasp ovipositor. By tuning the intensity, duration, spatial profile, and position of the laser focus, we could probe the conditions necessary for evoking calcium responses in class IV neurons, which were monitored by increases in GCaMP6f fluorescence under a spinning disk confocal microscope.

**Figure 7:**
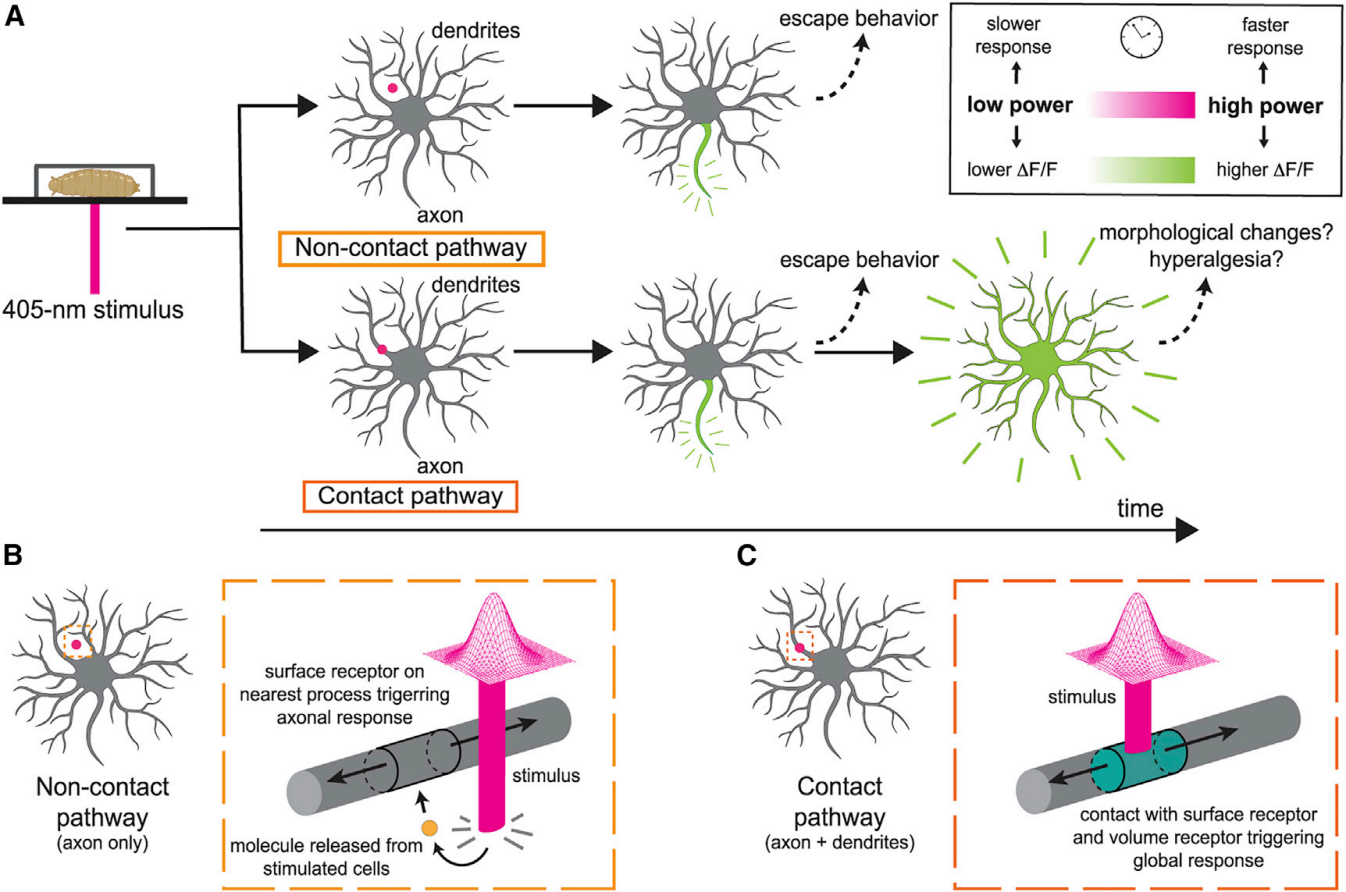
Summary of results and their interpretations. (*A*) Upper panels: noncontact stimulation (*magenta dot*) initiates axonal calcium responses. Lower panels: contact stimulation (*magenta dot*) initiates axonal and dendritic calcium responses. (*B*) Hypothetical mechanism underlying the noncontact response: damage to adjacent cells releases molecules (*orange circle*) that bind receptors on the dendritic surface, leading to cell depolarization. The depolarization is enough to trigger action potentials in the axon, which open calcium channels in the axon; the depolarization is insufficient to open calcium channels in the dendrites and soma. (*C*) Hypothetical mechanism underlying the contact response: direct damage to the dendrite strongly depolarizes the cell and opens calcium channels in dendrites, soma, and axon. The contact stimulus is also expected to also trigger the noncontact pathway. To see this figure in color, go online.

Our primary finding is that there are two distinct calcium signaling responses in class IV cells: 1) a noncontact response observed primarily in axons, and 2) a contact response seen in axons, dendrites, and cell bodies (Fig. 7 *A*). The existence of two response pathways is supported by three pieces of evidence: 1) axonal calcium signals do not require the laser spots to make direct contact with the dendritic processes (Fig. 2*, A and B*), whereas dendritic calcium signals require direct contact (Fig. 2*, C and D*); 2) axonal calcium signals are more sensitive and faster than dendritic calcium transients, even when the stimulus is as far as 400 *μ*m away from the axon (Figs. 3 and 4); and 3) the surface model provides a better fit to the axon responses, whereas the volume model provides a better fit to the dendrite and soma data (Fig. 6*, C–H*; Table S5). We believe that the noncontact response is due to localized mechanical damage, even at intensities less than 80%, in which there is no obvious puncture to the cuticle—although we suspect that there is damage to the surrounding cells that is not resolved by our imaging setup. An alternative hypothesis, namely that the calcium responses are due to delocalized photo-sensitive activation, is countered by the observation that wide-profile illumination generally gives smaller calcium responses (Fig. 4*, D–I*) despite delivering more power at larger distances (that could potentially directly stimulate the dendrite). Therefore, we argue that localized mechanical damage induced by the laser triggers noncontact responses in the axons and contact responses in all cellular compartments.

Given that stimulation with a focused laser shares several features with stimulation by an ovipositor—localized tissue damage, melanotic spots, behavioral responses, and axonal signals—we postulate that the ovipositor can excite the class IV neuron through both the contact and noncontact mechanisms. There are, however, some potential caveats to this conclusion. First, a wasp ovipositor punctures the cuticle via mechanical pressure, whereas our laser is likely damaging the cuticle via localized heating or production of reactive oxygen species by autofluorescence or GCaMP6f fluorescence. Second, although both the ovipositor and the focused laser produce localized damage, they are both expected to produce more delocalized effects on the tissue. The ovipositor is expected to generate a large strain field as the cuticle is indented before it ruptures. This strain field could evoke mechanoreceptive responses. The laser generates stray light over a wide area of the tissue through reflection and scattering, though the intensity is greatly attenuated. This stray light could excite photoreceptors (14) or the reactive oxygen species response (26). However, the stray light evidently does not excite dendritic calcium responses. This is likely because light alone is insufficient to induce calcium responses—instead, a localized wound is needed to initiate cellular calcium responses. Despite differences between laser and ovipositor stimulation and the considerable uncertainty about the precise effects of ovipositor penetration and laser illumination on the tissue, we believe that the ovipositor likely stimulates both contact and noncontact responses.

We propose the following pathways to account for the contact and noncontact calcium responses. First, we propose that direct contact of high-power laser illumination damages the class IV cell’s plasma membrane, making it more permeable to sodium and inducing a local depolarization of the membrane potential (2). The depolarization then spreads electrotonically throughout the dendrite to the cell body and the axon. Modeling electrotonic spread in the thin axons of primate rods and cones (which have diameters of 0.45 and 1.6 *μ*m, respectively) shows that there is little signal decrement over 400 *μ*m even at frequencies up to 50 Hz, which corresponds to a time constant <10 ms (27). Therefore, electrotonic spread of depolarization is likely fast enough to reach all parts of the class IV cell. If the depolarization exceeds the threshold needed to open L-type (and potentially other) calcium channels, then calcium will enter and a GCaMP6f fluorescent signal produced. If there are calcium channels in the dendrites, cell body, and axon, then fluorescence changes will be observed throughout the cell.

Second, we propose that if high-power laser illumination makes no contact with the class IV cell, it will, nevertheless, damage adjacent cells, such as the overlying epithelial (epidermal) cells and underlying muscle cells (6). These cells could then release small metabolites or acidify the extracellular space. These signals then spread by diffusion to the membrane of the class IV cells, where they open receptor-gated or the acid-sensing channels—for example, pickpocket or ripped pocket (28,29). This mechanism would be analogous to the release of cytosolic ATP from damaged cells, which mediates pain perception via contact with P2X receptors on peripheral nociceptive cells in vertebrates (19,20). Although *Drosophila* lacks P2X receptors (30), it is possible that other small cytoplasmic molecules or protons released by surrounding cells might play an analogous role. Opening of receptor-linked channels is expected to locally depolarize the cell membrane, and this depolarization will spread electrotonically to the cell body and axon, where if it exceeds a threshold, it leads to axonal action potentials, which in turn trigger the opening of calcium channels. If the receptor mechanism leads to less depolarization in class IV dendrites than direct damage, as is reasonable, then noncontact stimulation may be above threshold for action potentials in the axons (which then open calcium channels) but below threshold for opening calcium channels in the dendrites and soma. Hence, only axons respond to noncontact stimulation. Because direct contact is also likely to damage adjacent cells and trigger the noncontact response as well, axon responses are likely to be triggered by both pathways. Thus, there are likely two pathways by which localized damage by ovipositor barbs leads to electrophysiological and calcium responses.

Interestingly, the existence of these two pathways provides evidence that the dendrites of class IV cells are not electrically excitable. If they were excitable, then we would expect that axonal action potentials would back propagate and in turn stimulate calcium entry through voltage-gated channels in the dendrites, but the noncontact response does not stimulate calcium responses in dendrites. A related point is that when direct contact is made, the axonal calcium signals (Fig. S4) are usually more transient than the dendritic signals (Fig. S3). A possible explanation is that calcium entry opens calcium-activated potassium channels in the axons, which hyperpolarizes the axonal membrane tending to inhibit spiking and additional calcium entry. This delayed negative feedback would attenuate the calcium signal in the axon at longer times. The existence of axonal calcium-activated potassium channels could account for the “unconventional spikes” recorded from the cell body and the axon bundle (11); these spikes are characterized by an ensuing refractory period during which there is no spiking. The unconventional spikes and refractory period correlates with calcium signals in the dendrites and may be a consequence of the opening of calcium-activated potassium channels.

The existence of the noncontact pathway sheds new light on the highly branched morphology of class IV cells. Because the “mesh size”—the average distance between dendrites in the arbor—is ∼5 *μ*m, it has been suggested that the reason these cells are highly branched is to maximize direct contact with ovipositor barbs (18). However, the noncontact pathway implies that direct damage to the class IV cell is not necessary to stimulate the axonal pathway. However, the class IV cells still need to be highly branched and make a fine mesh so that extracellular signals can still diffuse sufficiently quickly to activate membrane receptors; a small molecule similar in size to ATP (diffusion coefficient on the order of 100 *μ*m^2^/s) will reach a dendrite 5 *μ*m away in ∼0.1 s. To diffuse a distance three times as far (15 *μ*m) would take ∼1 s, too slow to account for the axonal responses. Thus, our data lead us to propose a new function underlying extensive branching of class IV dendritic arbors; the fine meshwork minimizes diffusion times to ensure that noncontact stimulation is rapidly transduced.

Whereas the function of the axonal response is clear—to convey nociceptive signals to the central nervous system—the function of the dendrite response is not. The dendritic responses are often centrifugal, moving away from the cell body; they are therefore not on the cell-to-brain pathway. One implication is that dendritic calcium signals in class IV cells are not necessarily good proxies for neuronal excitation. Calcium signals are often assumed to be reporters of cell excitation, although a number of researchers have cautioned against this assumption (31,32). It is possible that dendritic calcium mediates hyperalgesia by modifying the sensitivity in case of a second attack. Alternatively, because severing dendrites leads to peripheral degeneration (33), it is possible that the dendrite-wide calcium signal could promote regrowth. These will be important possibilities to follow up on in future experiments.

## Author contributions

R.B. and J.H. conceptualized all experiments and models. R.B. performed all experiments. R.B. and S.S. performed all analysis. R.B., S.S., and J.H. prepared the manuscript.

## Supporting material

Document S1. Figs. S1–S5 and Tables S1–S11

Document S2. Article plus supporting material
