# Supplementary material for: Focal laser stimulation of fly nociceptors activates distinct axonal and dendritic Ca2+ signals
Source: Biophys J. 2021 Jun 25;120(15):3222–33. doi: 10.1016/j.bpj.2021.06.001 (PMC8390926; doi:10.1016/j.bpj.2021.06.001)
Supplement: Document S1. Figs. S1–S5 and Tables S1–S11 [file mmc1.pdf]

Biophysical Journal, Volume *120*

Supplemental information

Focal laser stimulation of fly nociceptors activates distinct axonal and dendritic Ca^{2+} signals

Rajshekhar Basak, Sabyasachi Sutradhar, and Jonathon Howard

Focal Laser Stimulation of Fly Nociceptors Activates Distinct Axonal and Dendritic Ca^{2+} Signals

Supplementary Materials

Rajshekhar Basak[1], Sabyasachi Sutradhar[1] and Jonathon Howard[1]

[1]Department of Molecular Biophysics & Biochemistry,

Yale University, New Haven, CT 06511

A

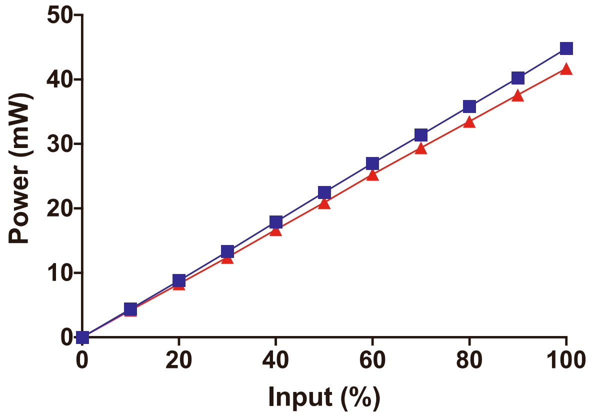

Supplementary Figure 1: Measured power output (mW) across various input values (%) from 405-nm stimulus used to pulse Class IV neurons. Blue corresponds to stimulus with FWHM = 0.5 μm, red corresponds to stimulus with FWHM = 1 μm. Measurements were made using a microscope slide power sensor (S170C, Thor Labs) and a Touchscreen Optical Power and Energy Meter Console (PM400, Thor Labs) at the sample plane.

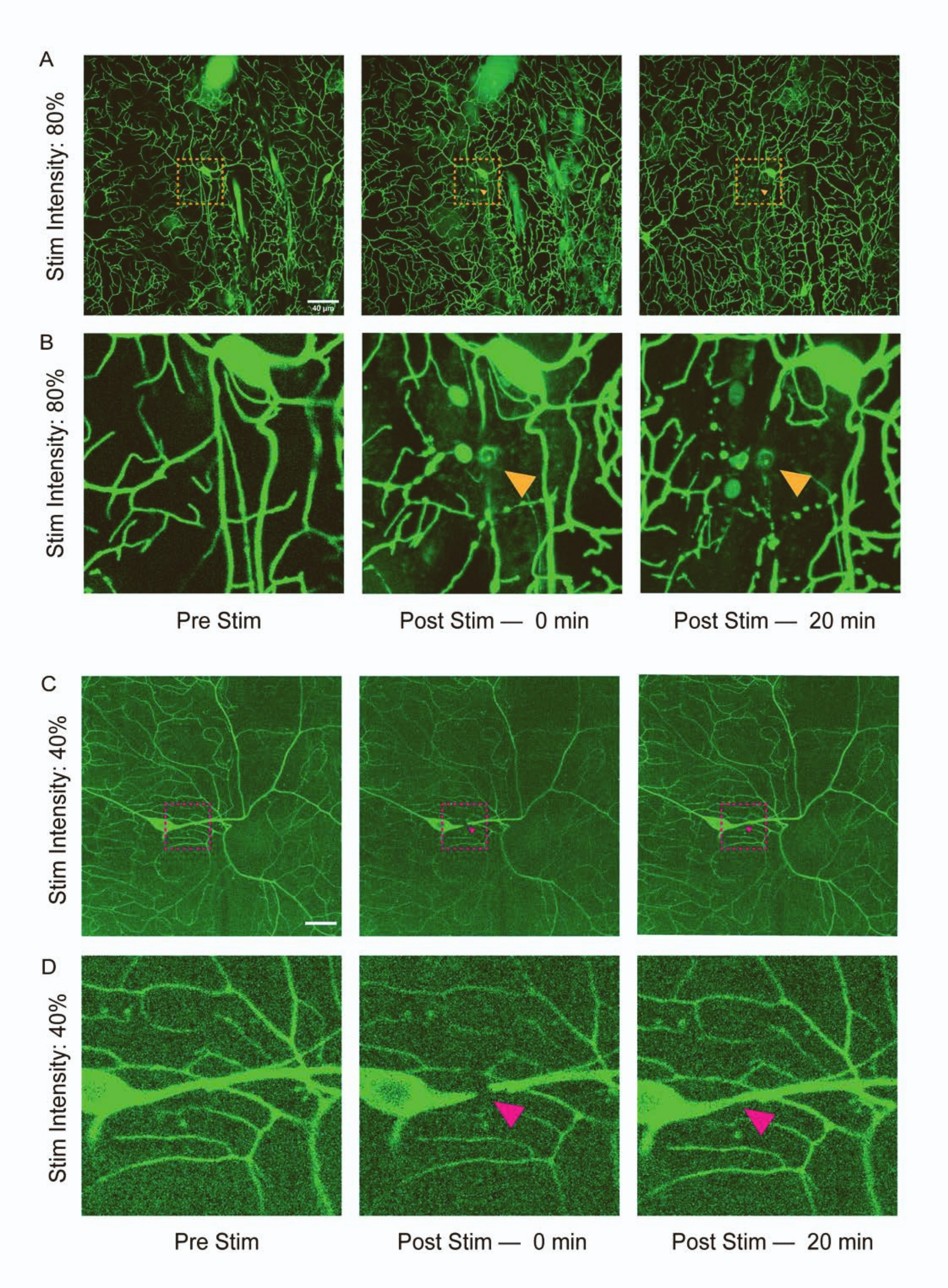

Supplementary Figure 2: 405-nm stimulation causes bleaching versus puncture of larval cuticle depending on the wattage delivered. Panels shown are pre-stim (left), frame immediately following stimulation (center), and 20 minutes post-stimulation (right). (A) Montage of Class IV da neurons expressing CD4-td-GFP stimulated with 405-nm laser at 80% power. (B) Zoom-in of region indicated by the dashed magenta box shown in panel (A). Orange arrow highlights region where laser was focused. Central and right panels show puncture wound that does not recover (not photobleaching) 20 minutes post stimulation. (C) Montage of Class IV da neurons expressing CD4-td-GFP stimulated with 40- nm laser at 40% power. (D) Zoom-in of region indicated by the dashed magenta box shown in (C). Magenta arrow indicates region where laser was focused. Central panel shows localized bleaching of dendritic process; right panel shows same region after recovery (20 minutes post stimulation) with no puncture wound.

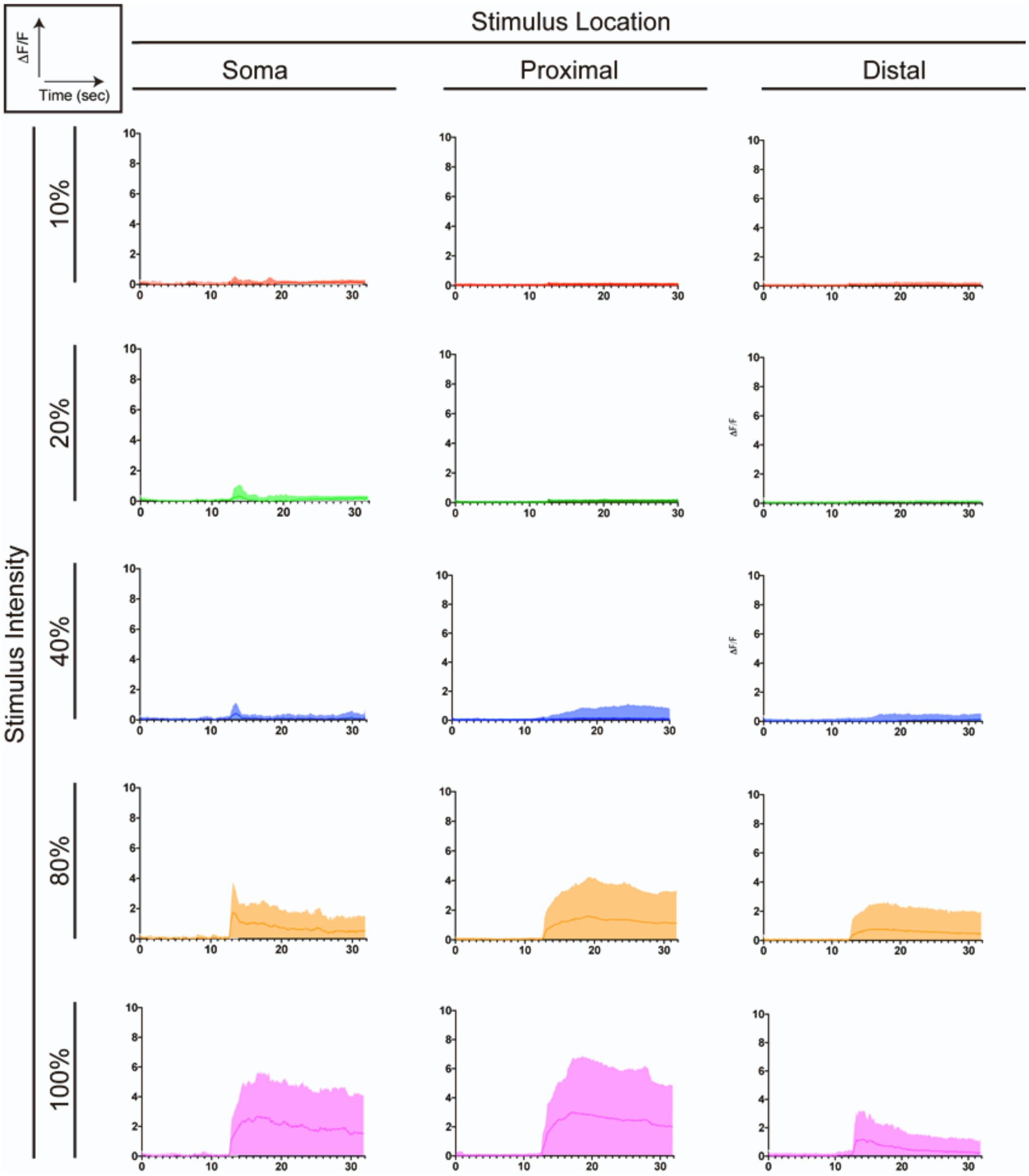

Supplementary Figure 3: Representative $\Delta F/F$ traces of dendrite and soma ROIs for varying stimulation locations and wattages. Stimulus was activated at the 12.43 second mark (100th frame). Data across all experiments were combined. Mean and SD shown.

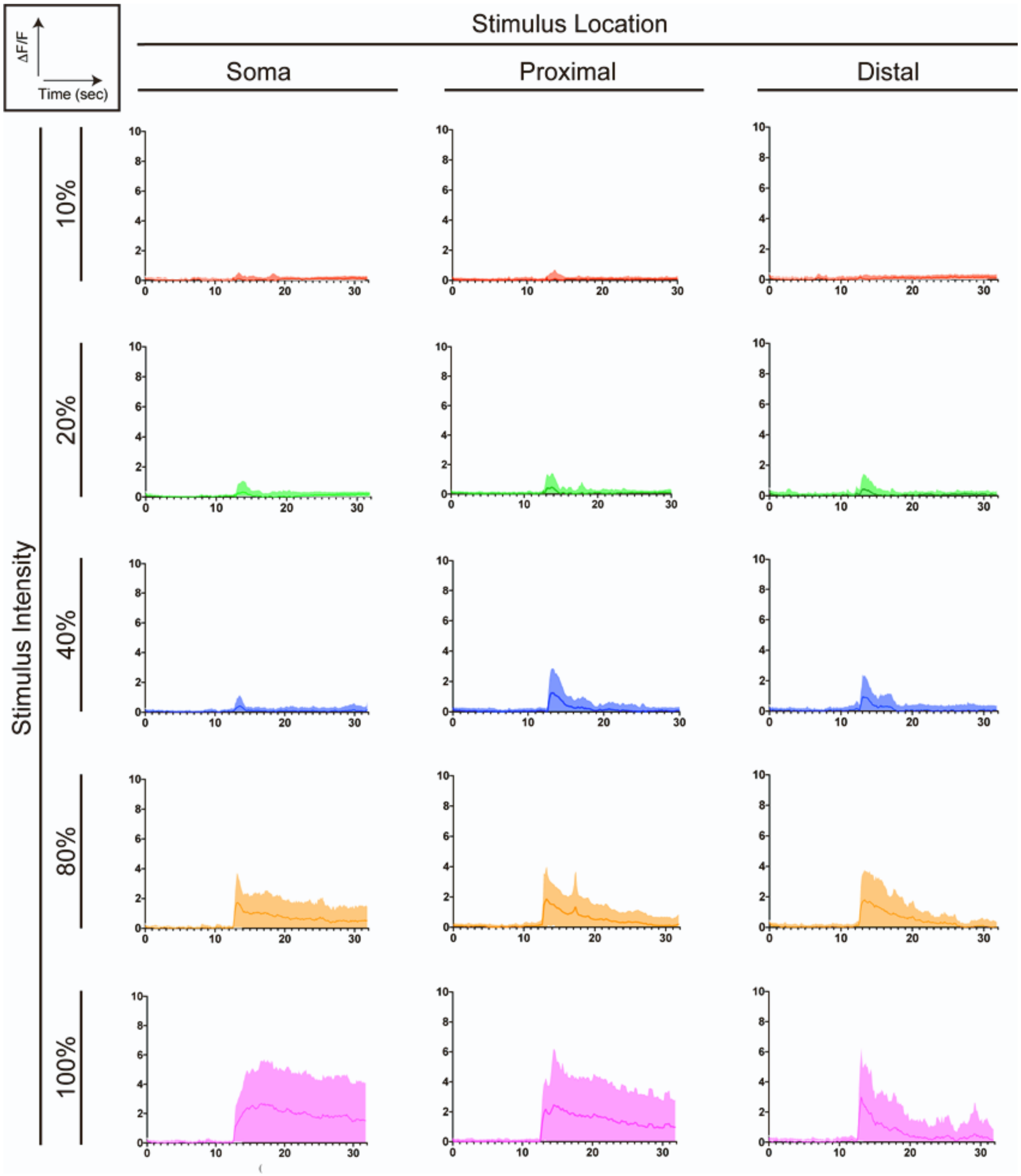

Supplementary Figure 4: Representative $\Delta F/F$ traces of axon ROIs for varying stimulation locations and wattages. Stimulus was activated at the 12.43 second mark (100th frame). Data across all experiments were combined. Mean and SD shown

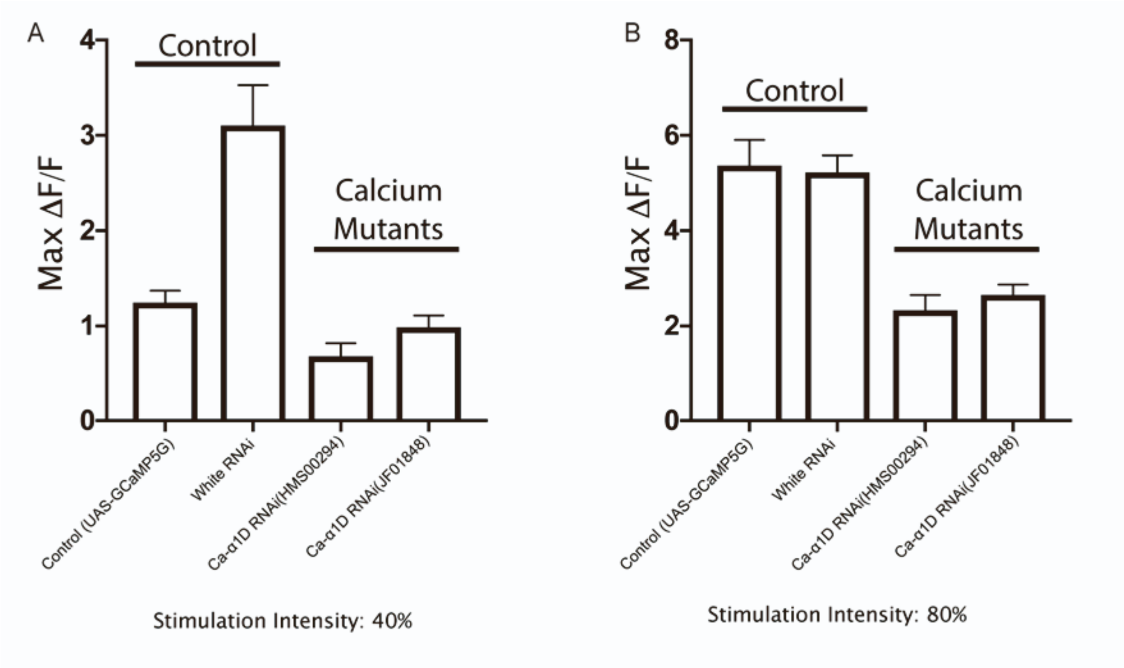

Supplementary Figure 5: Magnitudes of calcium transients from control (; UAS-GCaMP5G / ppk-GAL4; | N = 24, n = 72 and ; ppk-GAL4/+ ; white RNAi / UAS-GCaMP5G | N = 24, n = 72) and mutant cells (; ppk-GAL4/ + ; *Ca-α1D RNAi* ^{JF01848} / UAS-GCaMP5G | N = 24, n = 72 and ; ppk-GAL4/+ ; *Ca-α1D RNAi* ^{HMS00294}/ UAS-GCaMP5G | N = 24, n = 72) irradiated at two different stimulation intensities, 40% (A) and 80% (B). Plots show mean and SEM.

	Fig 3C, 4D	Fig 3D, 4G
	**Axon (green) — "Non-contact" (FWHM: 1 µm)** vs. **Axon (green) — "Contact" (soma) (FWHM: 1 µm)**	**Axon (green) — "Non-contact" (FWHM: 0.5 µm)** vs. **Axon (green) — "Contact" (soma) (FWHM: 0.5 µm)**
**10%**	$p > 0.9999$ (ns)	$p > 0.9999$ (ns)
**20%**	$p > 0.9999$ (ns)	$p > 0.9999$ (ns)
**40%**	$p = 0.9995$ (ns)	$p > 0.9999$ (ns)
**80%**	$p > 0.9999$ (ns)	$p < 0.0001$ (****) (Contact stimulation gives larger response magnitude than non-contact)
**100%**	$p = 0.0954$ (ns)	$p < 0.0003$ (***) (Contact stimulation gives larger response magnitude than non-contact)

Supplementary Table 1: Comparison of magnitude of axon responses resulting from "non-contact" and "contact" stimulation (green ROIs and line) as shown in Fig 3C,D (non-contact stimulation) and Fig 4D,G (**soma** stimulation). Comparisons are shown for stimulation with FWHM: 1 µm and FWHM: 0.5 µm. Multiplicity adjusted p-values computed using Sidak's multiple comparisons test shown (performed using Prism 8).

	Fig 3C, 4E	Fig 3D, 4H
	**Axon (green) — "Non-contact" (FWHM: 1 µm)** vs. **Axon (green) — "Contact" (prox) (FWHM: 1 µm)**	**Axon (green) — "Non-contact" (FWHM: 0.5 µm)** vs. **Axon (green) — "Contact" (prox) (FWHM: 0.5 µm)**
**10%**	$p > 0.9999$ (ns)	$p > 0.9999$ (ns)
**20%**	$p > 0.9999$ (ns)	$p > 0.9999$ (ns)
**40%**	$p > 0.9999$ (ns)	$p > 0.9999$ (ns)
**80%**	$p > 0.9999$ (ns)	$p < 0.1381$ (ns)
**100%**	$p = 0.8005$ (ns)	$p < 0.0001$ (****) (Contact stimulation gives larger response magnitude than non-contact)

Supplementary Table 2: Comparison of magnitude of axon responses resulting from "non-contact" and "contact" stimulation (green ROIs and line) as shown in Fig 3C,D (non-contact stimulation) and Fig 4E,H (**proximal** stimulation). Comparisons are shown for stimulation with FWHM: 1 µm and FWHM: 0.5 µm. Multiplicity adjusted p-values computed using Sidak's multiple comparisons test shown (performed using Prism 8).

	Fig 3C, 4F	Fig 3D, 4I
	**Axon (green) — “Non-contact” (FWHM: 1 µm)** vs. **Axon (green) — “Contact” (distal) (FWHM: 1 µm)**	**Axon (green) — “Non-contact” (FWHM: 0.5 µm)** vs. **Axon (green) — “Contact” (distal) (FWHM: 0.5 µm)**
**10%**	$p > 0.9999$ (ns)	$p > 0.9999$ (ns)
**20%**	$p > 0.9999$ (ns)	$p > 0.9999$ (ns)
**40%**	$p > 0.9999$ (ns)	$p > 0.9999$ (ns)
**80%**	$p > 0.9999$ (ns)	$p > 0.9999$ (ns)
**100%**	$p = 0.9882$ (ns)	$p > 0.9999$ (ns)

Supplementary Table 3: Comparison of magnitude of axon responses resulting from “non-contact” and “contact” stimulation (green ROIs and line) as shown in Fig 3C,D (non-contact stimulation) and Fig 4F,I (**distal** stimulation). Comparisons are shown for stimulation with FWHM: 1 µm and FWHM: 0.5 µm. Multiplicity adjusted p-values computed using Sidak's multiple comparisons test shown (performed using Prism 8).

	Figure 4D,G<br><br>Soma Stim	Figure 4D,G<br><br>Prox Stim	Figure 4E,H<br><br>Distal Stim	Figure 4E,H<br><br>Soma Stim	Figure 4F,I<br><br>Prox Stim	Figure 4F,I<br><br>Distal Stim
	**Axon (green line) (FWHM: 1 μm)** vs. **Axon (green line) (FWHM: 0.5 μm)**	**Axon (green line) (FWHM: 1 μm)** vs. **Axon (green line) (FWHM: 0.5 μm)**	**Axon (green line) (FWHM: 1 μm)** vs. **Axon (green line) (FWHM: 0.5 μm)**	**Dendrite & Soma (black line) (FWHM: 1 μm)** vs. **Dendrite & Soma (black line) (FWHM: 0.5 μm**	**Dendrite & Soma (black line) (FWHM: 1 μm)** vs. **Dendrite & Soma (black line) (FWHM: 0.5 μm)**	**Dendrite & Soma (black line) (FWHM: 1 μm)** vs. **Dendrite & Soma (black line) (FWHM: 0.5 μm)**
**10%**	p = 0.9998<br><br>(ns)	p > 0.9999<br><br>(ns)	p > 0.9999<br><br>(ns)	p > 0.9999<br><br>(ns)	p = 0.9998<br><br>(ns)	p > 0.9999<br><br>(ns)
**20%**	p > 0.9999<br><br>(ns)	p = 0.9999<br><br>(ns)	p > 0.9999<br><br>(ns)	p = 0.8078<br><br>(ns)	p > 0.9999<br><br>(ns)	p = 0.0675<br><br>(ns)
**40%**	p = 0.2251<br><br>(ns)	p = 0.9626<br><br>(ns)	p = 0.9434<br><br>(ns)	p = 0.1265<br><br>(ns)	p = 0.0605<br><br>(ns)	p > 0.9999<br><br>(ns)
**80%**	p < 0.0001<br><br>(****)	p < 0.0563<br><br>(ns)	p > 0.9999<br><br>(ns)	p < 0.0001<br><br>(****)	p = 0.0016<br><br>(**)	p < 0.0001<br><br>(****)
**100%**	p = 0.0746<br><br>(ns)	p = 0.0012<br><br>(**)	p = 0.9940<br><br>(ns)	p < 0.0001<br><br>(****)	p = 0.9380<br><br>(ns)	p < 0.0001<br><br>(****)

Supplementary Table 4: Comparison of magnitude of calcium responses shown in Figure 4 D-I across two different stimulation irradiance settings. Axon ROI (FWHM: 1 μm) were compared to axon ROI (FWHM: 0.5 μm). Dendrite & soma ROI (FWHM: 1 μm) were compared to dendrite & soma ROI (FWHM: 0.5 μm). Multiplicity adjusted p-values computed using Sidak's multiple comparisons test shown (performed using Prism 8).

	**Surface Model** *(Equation 1)*			**Volume Model** *(Equation 2)*		
	**n**	γ	**Error**	**n**	γ	**Error**
**Dendrites and Soma ROI**	1.71	8.125	56.99	1.87	0.097	39.07
**Axon ROI**	1.33	0.346	24.29	1.66	0.015	42.19

Supplementary Table 5: Summary of modeling fit parameters for dendrite and soma ROI and axon ROIs using the surface model (equation 1, *See Materials and Methods*) and volume model (equation 2, *See Materials and Methods*). Input parameters for the model are provided separately in Table 6 and Table 7. Visual plots of modeling results are shown in Figure 6 C-H.

	Figure 3C	Figure 3C	Figure 3C, D	Figure 3C, D
	**Axon (green line) (FWHM: 1 μm)** vs. **Dendrite & Soma (black line) (FWHM: 1 μm)**	**Axon (green line) (FWHM: 0.5 μm)** vs. **Dendrite & Soma (black line) (FWHM: 0.5 μm)**	**Axon (green line) (FWHM: 1 μm)** vs. **Axon (green line) (FWHM: 0.5 μm)**	**Dendrite & Soma (black line) (FWHM: 1 μm)** vs. **Dendrite & Soma (black line) (FWHM: 0.5 μm)**
**10%**	p = 0.9317 (ns)	p = 0.1117 (ns)	p = 0.9751 (ns)	p = 0.8456 (ns)
**20%**	P = 0.7630 (ns)	p = 0.0175 (*)	p = 0.7790 (ns)	p = 0.9719 (ns)
**40%**	p < 0.0001 (****)	p < 0.0001 (****)	p = 0.9896 (ns)	p = 0.1417 (ns)
**80%**	p < 0.0001 (****)	p < 0.0001 (****)	p >0.9999 (ns)	p = 0.0032 (**)
**100%**	p < 0.0001 (****)	p < 0.0001 (****)	p >0.9999 (ns)	p = 0.0193 (**)

Supplementary Table 6: Comparison of magnitude of calcium responses shown in Figure 3 C, D. Multiplicity adjusted p-values computed using Sidak's multiple comparisons test shown (performed using Prism 8).

	**Figure 3E**
	**Axonal Response latency** **(FWHM: 1 µm)** vs. **(FWHM: 1 µm)**
**10%**	p = 0.2011 (ns)
**20%**	p = 0.0004 (***)
**40%**	p = 0.4851 (ns)
**80%**	p = 9710 (ns)
**100%**	p > 0.9999 (ns)

Supplementary Table 7: Comparison of latencies for axon ROIs stimulated under the “non-contact” condition as shown in Figure 3E. Multiplicity adjusted p-values computed using Sidak's multiple comparisons test shown (performed using Prism 8).

	Figure 4D<br>Soma Stim	Figure 4E<br>Prox Stim	Figure 4F<br>Distal Stim	Figure 4G<br>Soma Stim	Figure 4H<br>Prox Stim	Figure 4I<br>Distal Stim
	**Axon (green line) (FWHM: 1 µm) vs. Dendrite & Soma (black line) (FWHM: 1 µm)**	**Axon (green line) (FWHM: 1 µm) vs. Dendrite & Soma (black line) (FWHM: 1 µm)**	**Axon (green line) (FWHM: 1 µm) vs. Dendrite & Soma (black line) (FWHM: 1 µm)**	**Axon (green line) (FWHM: 0.5 µm) vs. Dendrite & Soma (black line) (FWHM: 0.5 µm)**	**Axon (green line) (FWHM: 0.5 µm) vs. Dendrite & Soma (black line) (FWHM: 0.5 µm)**	**Axon (green line) (FWHM: 0.5 µm) vs. Dendrite & Soma (black line) (FWHM: 0.5 µm)**
**10%**	p = 0.9975 (ns)	p = 0.9998 (ns)	p = 0.9998 (ns)	p > 0.9999 (ns)	p > 0.9999 (ns)	p > 0.9999 (ns)
**20%**	p = 0.8916 (ns)	p = 0.9006 (ns)	p = 0.6027 (ns)	p = 0.9993 (ns)	p = 0.9991 (ns)	p = 0.9904 (ns)
**40%**	p = 0.9993 (ns)	p = 0.2950 (ns)	p = 0.0041 (**)	p > 0.9999 (ns)	p = 0.6824 (ns)	p = 0.2377 (ns)
**80%**	p = 0.9981 (ns)	p = 0.9921 (ns)	p = 0.0010 (***)	p = 0.9593 (ns)	p = 0.7610 (ns)	p = 0.9954 (ns)
**100%**	p = 0.4851 (ns)	p = 0.3031 (ns)	p = <0.0001 (****)	p = 0.5376 (ns)	p = 0.8493 (ns)	p > 0.9999 (ns)

Supplementary Table 8: Comparison of magnitude of calcium responses between axons and dendrite/soma ROIs for the “contact” dendritic response as shown in Figures 4 D-I. Multiplicity adjusted p-values computed using Sidak's multiple comparisons test shown (performed using Prism 8).

	Figure 5A, B	Figure 5A, B
	**Dendrite & Soma (black)** (FWHM: 1 μm) vs. **Dendrite & Soma (black)** (FWHM: 0.5 μm)	**Axon (green)** (FWHM: 1 μm) vs. **Axon (green)** (FWHM: 0.5 μm)
**10%**	$p = 0.0052$ (**)	$p = 0.9994$ (ns)
**20%**	$p < 0.0001$ (****)	$p > 0.9999$ (ns)
**40%**	$p < 0.0001$ (****)	$p > 0.9999$ (ns)
**80%**	$p = 0.9046$ (ns)	$p > 0.9999$ (ns)
**100%**	$p = 0.9537$ (ns)	$p > 0.9999$ (ns)

Supplementary Table 9: Comparison of latencies for ROIs stimulated with two different irradiance settings in Figure 5. Multiplicity adjusted p-values computed using Sidak's multiple comparisons test shown (performed using Prism 8).

Power (P) (%)	Laser Width (σ) (nm)	Dendrite Radius (r) (nm)	Experimental $\Delta F/F$ (F)	Modeled SEM (WLS weight)
0	212.31	500	0	0.1558
10	212.31	500	0.182350664	0.22717205
20	212.31	500	0.076460389	0.185726596
40	212.31	500	2.050158743	0.958232132
80	212.31	500	5.025163541	2.12264901
100	212.31	500	5.074426106	2.141930378
0	212.31	200	0	0.1558
10	212.31	200	0.005188064	0.157830608
20	212.31	200	0.860460567	0.492584266
40	212.31	200	0.164808405	0.22030601
80	212.31	200	2.548603702	1.153323489
100	212.31	200	2.948527166	1.309853533
0	212.31	1000	0	0.1558
10	212.31	1000	0.03473628	0.16939578
20	212.31	1000	0.763529255	0.45464535
40	212.31	1000	1.779650087	0.852355044
80	212.31	1000	4.722394194	2.004145087
100	212.31	1000	7.703064753	3.170779545
0	424.62	500	0	0.1558
10	424.62	500	0.008014336	0.158936811
20	424.62	500	0	0.1558
40	424.62	500	0.182200981	0.227113464
80	424.62	500	2.328810344	1.067296369
100	424.62	500	4.482075196	1.910084232
0	424.62	200	0	0.1558
10	424.62	200	0.022429348	0.164578847
20	424.62	200	0.008520127	0.159134778
40	424.62	200	0.163382325	0.219747842
80	424.62	200	0.892799903	0.505241882
100	424.62	200	1.369965549	0.692004516
0	424.62	1000	0	0.1558
10	424.62	1000	0.003978684	0.157357257
20	424.62	1000	0.076913166	0.185903813
40	424.62	1000	0.366020076	0.299060258
80	424.62	1000	1.869151273	0.887385808
100	424.62	1000	4.580236882	1.948504716

Supplementary Table 10: Modeling input parameter values for dendrite and soma ROI. 100% power corresponds to 43 mW for both laser profiles (see Supplementary Figure 1).

Power (P) (%)	Laser Width (σ) (nm)	Dendrite Radius (r) (nm)	Experimental $\Delta F/F$ (F)	Modeled SEM (WLS weight)
0	212.31	500	0	0.1445
10	212.31	500	0.068684925	0.168677094
20	212.31	500	0.392383484	0.282618986
40	212.31	500	0.782529208	0.419950281
80	212.31	500	3.872628207	1.507665129
100	212.31	500	6.07809914	2.283990897
0	212.31	200	0	0.1445
10	212.31	200	0.01168088	0.14861167
20	212.31	200	0.43328868	0.297017615
40	212.31	200	1.77380998	0.768881113
80	212.31	200	2.18446697	0.913432373
100	212.31	200	2.81976898	1.137058681
0	212.31	1000	0	0.1445
10	212.31	1000	0	0.1445
20	212.31	1000	0.360228787	0.271300533
40	212.31	1000	2.011095156	0.852405495
80	212.31	1000	5.684920583	2.145592045
100	212.31	1000	5.734179428	2.162931159
0	424.62	500	0	0.1445
10	424.62	500	0.140546121	0.193972235
20	424.62	500	0.54426786	0.336082287
40	424.62	500	1.292631317	0.599506224
80	424.62	500	2.027996352	0.858354716
100	424.62	500	3.37835648	1.333681481
0	424.62	200	0	0.1445
10	424.62	200	0.091146399	0.176583532
20	424.62	200	0.429240122	0.295592523
40	424.62	200	1.189055302	0.563047466
80	424.62	200	2.035088751	0.86085124
100	424.62	200	3.170241314	1.260424942
0	424.62	1000	0	0.1445
10	424.62	1000	0.182453729	0.208723713
20	424.62	1000	0.498731771	0.320053583
40	424.62	1000	0.500696023	0.320745
80	424.62	1000	2.037007512	0.861526644
100	424.62	1000	3.852882468	1.500714629

Supplementary Table 11: Modeling input parameter values for axon ROIs. 100% power corresponds to 43 mW for both laser profiles (see Supplementary Figure 1).
